# Agent-Based Model of Human Alveoli Predicts Chemotactic Signaling by Epithelial Cells during Early *Aspergillus fumigatus* Infection

**DOI:** 10.1371/journal.pone.0111630

**Published:** 2014-10-31

**Authors:** Johannes Pollmächer, Marc Thilo Figge

**Affiliations:** 1 Applied Systems Biology, Leibniz-Institute for Natural Product Research and Infection Biology, Hans Knöll Institute, Jena, Germany; 2 Friedrich Schiller University, Jena, Germany; Louisiana State University, United States of America

## Abstract

*Aspergillus fumigatus* is one of the most important human fungal pathogens, causing life-threatening diseases. Since humans inhale hundreds to thousands of fungal conidia every day, the lower respiratory tract is the primary site of infection. Current interaction networks of the innate immune response attribute fungal recognition and detection to alveolar macrophages, which are thought to be the first cells to get in contact with the fungus. At present, these networks are derived from *in vitro* or *in situ* assays, as the peculiar physiology of the human lung makes *in vivo* experiments, including imaging on the cell-level, hard to realize. We implemented a spatio-temporal agent-based model of a human alveolus in order to perform *in silico* experiments of a virtual infection scenario, for an alveolus infected with *A. fumigatus* under physiological conditions. The virtual analog captures the three-dimensional alveolar morphology consisting of the two major alveolar epithelial cell types and the pores of Kohn as well as the dynamic process of respiration. To the best of our knowledge this is the first agent-based model of a dynamic human alveolus in the presence of respiration. A key readout of our simulations is the first-passage-time of alveolar macrophages, which is the period of time that elapses until the first physical macrophage-conidium contact is established. We tested for random and chemotactic migration modes of alveolar macrophages and varied their corresponding parameter sets. The resulting first-passage-time distributions imply that randomly migrating macrophages fail to find the conidium before the start of germination, whereas guidance by chemotactic signals derived from the alveolar epithelial cell associated with the fungus enables a secure and successful discovery of the pathogen in time.

## Introduction

*Aspergillus fumigatus* is the most frequently isolated invasive mould in clinical patients [1]. With an increasing number of severe infections over the last two decades this ubiquitous saprophytic fungus has become one of the most important fungal pathogens [2]. The fungus exhibits its pathogenic behaviour primarily in immunosuppressed individuals, leading to life-threatening diseases with invasive aspergillosis being the most lethal one [3], [4]. Depending on the environmental conditions humans inhale several hundreds up to thousands of conidia of *A. fumigatus* every day [5]. Due to their small size of about 2–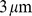 in diameter, a fraction of the inhaled conidia is able to evade the filter activity of the human lung and reaches the alveoli [6], [7]. Here, conidia are controlled and eliminated predominantly by innate immune responses [8].

Alveolar macrophages (AM) are professional phagocytes of the innate immune system resident in alveoli, where they trigger immune-responses to a vast amount of inhaled substances and particles [9]. They are located on the surface of alveolar epithelial cells (AEC) and shielded from direct contact to the alveolar airspace by the thin surfactant film [10]. AM are able to detect and phagocytose resting and swollen conidia of *A. fumigatus*, whereas the swollen phenotype is a prerequisite for killing [8]. Typical *in vitro* assays of host-pathogen interaction use high cell densities [11], [12], *i.e.* at any time phagocytes and pathogens are in close range and these unphysiological conditions render the experiments unrealistic with regard to the active search of phagocytes for pathogens. Similarly, animal models of aspergillus infection deal with the application of several millions of conidia in one shot in order to measure survival rates [12], [13]. Such approaches do not reflect the actual physiological conditions under which aspergillosis develops and consequently important aspects of the host response, such as the dynamics of phagocytes searching for pathogens, are obscured. Due to the peculiar physiology of the human lung, various measures are also not directly accessible *in vivo*. For example, the migration mode and speed of AM could not be investigated today *in vivo*. *In vitro* studies of AM motility [10], [11] generally revealed low speeds of AM with an average value of four micrometer per minute under chemotactic conditions [14]. Nevertheless, it may well be that AM present their true migration activity only in their natural environment.

By means of agent-based modelling and computer simulation we are in the position to study the early immune response in human alveoli by establishing a virtual infection model in space and time. This is similar to the virtual infection model for *Candida albicans* in human blood that was recently established by [15] to quantify immune effector mechanisms. Two important aspects regarding the early immune response upon the encounter with *A. fumigatus* have been investigated *in silico*. One aspect of phagocyte activity is the search strategy allowing to rapidly find the pathogen and to efficiently clear the infection. This was studied before by [16] and [17] using agent-based modelling. Tokarski *et al.* compared the performance of various search strategies by measuring the amount of pathogens that were phagocytozed by a large number of phagocytes in the course of time, whereas Charnick *et al.* investigated single phagocyte-pathogen encounters for varying search strategies. Both models comprise active phagocyte migration with and without chemotactic signalling on a two-dimensional grid representing the surface of the lung, but do not account for the three-dimensional morphology and peculiar boundary conditions of alveoli.

In this study, we put special emphasis on the spatio-temporal reconstruction of the human alveolus, including the spherical morphology [18], the composition of the alveolar surface by AEC type I and type II cells [19] and the pores of Kohn, representing connections between adjacent alveoli [20]. We investigate the dynamics of the alveolus under typical respiration conditions and for potential active migration modes of AM during the early immune response. An important readout of our simulations is the *first-passage time* (FPT), *i.e.* the time required for a phagocyte to find a pathogen in this complex biological structure. It is well known that analytical solutions for the FPT probability distribution can only be obtained for relatively simple systems with low spatial dimensions, whereas for higher spatial dimensions and complex geometries only the mean value of the FPT may be analytically tractable [21]. Therefore, computer simulations are required to numerically retrieve the distribution of FPT in the agent-based model. In combination with typical times of *A. fumigatus* germination, it is possible to assess the migration behaviour of AM with regard to infection clearance. Our results clearly indicate that random migration of AM is not an adequate type of migration for searching alveoli and detecting conidia before germination starts. In order to realize this in time, chemotactic signals are required that guide AM toward positions of conidia and we propose how this prediction could be tested in experiment.

## Model

A spatio-temporal agent-based model is implemented to perform *in silico* experiments on first-passage-time (FPT) measurements for *A. fumigatus* conidia being found by AM in alveoli. We take advantage of a cell-scale model, where each single cell of the system is represented by one discrete entity in the computer. In what follows, we present a detailed description of each cell-type, *i.e.* its static and dynamic attributes, as well as of the biological environment.

### Setup of the alveolus

**Figure 1:**
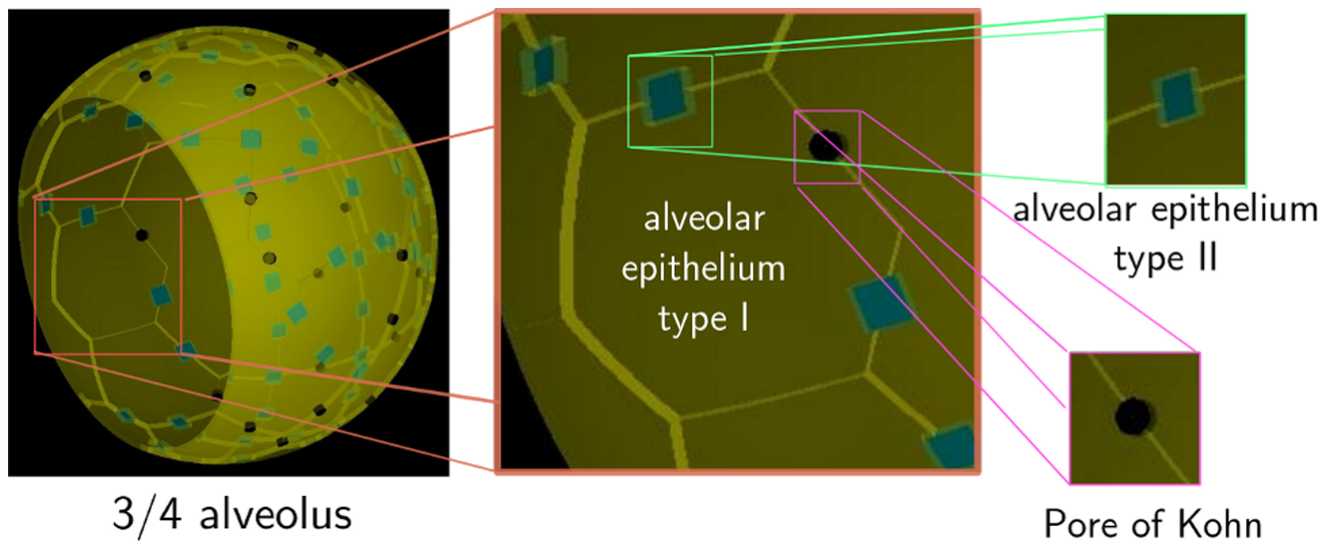
Three-dimensional reconstruction of the human three-quarter alveolus. Based on a literature survey (parameters are shown in Table 1) we reconstructed the spatial environment of one typical human alveolus, including the spherical shape of the system, the two major AEC types present in alveoli and the pores of Kohn as interalveolar connections (see Video S1).

The three-dimensional spatial environment of the model represents a typical human alveolus. The morphology of alveoli is polyhedral, resulting in a large surface-to-volume ratio in comparison to a sphere [10]. Previous *in silico* models, e.g. for aerosol transport and particle deposition in the human lung [22], were successfully based on a sphere-approximation of alveoli. A geometrical structure with spherical symmetry is most convenient for computational simulation models as the surface can be mathematically represented using spherical coordinates and was also used in the present study. We consider the most frequent case in human beings, corresponding to a three-quarter spherical alveolus [18] of radius 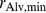 in the deflated state (see Fig. 1 and Video S1). The alveolus is constructed from a full sphere with surface points 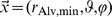 that is reduced to a three-quarter sphere regarding the volume fraction by using a lower threshold 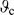 of the polar angle 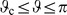. The alveolar entrance ring is defined by the positions 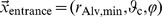 with 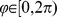.

Based on a literature survey on alveolar ducts (see Table 1), we reconstruct the cell-types belonging to the alveolar epithelium and the pores of Kohn, which represent inter-alveolar connections between neighbouring alveoli (see Fig. 1). Cell-numbers are estimated from the proportions of surface coverage and their respective sizes. To realize a realistic construction of the alveolus we proceed as follows. We first place centroids of type I AEC on the surface of the three-quarter sphere in a close-to-equidistant fashion (see Text S1 further details). Next, a Voronoi tesselation based on these centroids is projected onto the spherical surface of the alveolus to obtain type I AEC on the curved geometry as depicted in Fig. 1. Alveolar epithelial type II cells have a cuboidal shape and are randomly placed at the edges of neighbouring type I cells. A similar random procedure is used for positioning pores of Kohn with cylindrical shape (see Fig. 1). Note that pores of Kohn as well as the alveolar entrance ring represent open boundaries of the system through which AM can enter or exit the alveolus. The distributed open boundaries of the system strongly contribute to the complexity of alveoli.

**Table 1:** Model parameters for entities in the human alveolus.

parameter	description	value	comments
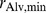	radius of one alveolus in deflated state	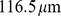	[22]
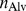	overall number of alveoli in adult human	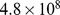	[36]
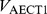	volume of alveolar epithelial type I cells	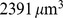	[37]
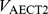	volume of alveolar epithelial type II cells	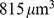	[37]
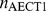	number of alveolar epithelial type I per alveolus	39–45	estimation
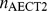	number of alveolar epithelial type II per alveolus	74–84	estimation
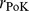	radius of one pore of Kohn	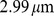	[38], estimation
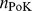	number of pores of Kohn per alveolus	24	[38]

The alveolus with constant radius 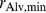 is extended by the implementation of two respiration modes, referred to as *resting condition* and *heavy exercise*, that differ in the respiration frequencies and spherical maximum radii (see Table 2 for values). The volume change of the alveolus by inflation and deflation during each respiration cycle is assumed to occur with equal time intervals for inspiration and expiration. We incorporate the increase and the decrease of the three-quarter sphere by periodically changing its time-dependent radius 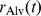 according to a piecewise linear function for the gain and the loss in the alveolar volume:
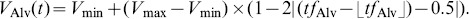
(1)Here, 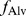 denotes the respiration frequency and 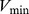 and 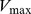 are the volumes of the three-quarter sphere in the deflated and inflated state, respectively.

**Table 2:** Model parameters for the static case and the two breathing conditions.

		values			
			resting	heavy	
parameter	description	static	condition	exercise	unit
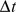	global timestep of the simulation	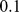	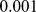	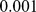	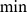
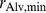	radius of one alveolus in deflated state	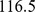	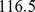	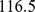	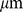
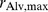	radius of one alveolus in expanded state	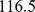	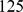	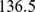	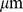
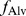	frequency of respiration	0	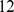	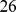	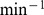
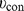	turbulence speed of *A. fumigatus*	0	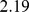	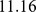	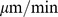
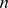	number of simulation runs per parameter-set	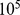	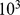	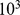	

### Passive movement of *A. fumigatus* conidia

We account for the passive movement of conidia that is induced by the contraction and expansion of the alveolus due to respiration. This is modeled by random walk migration with a speed that depends on the difference between the minimum radius 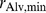 and the maximum radius 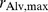 of the alveolus, on the frequency of respiration 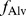 and on the radius of the conidium 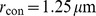:
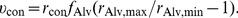
(2)This speed value assures that - in going from the deflated to the inflated state of the alveolus - the displacement 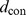 of the conidium during one respiratory cycle does not exceed the maximum value 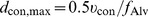. As follows from the theorem of intersecting lines, 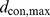 corresponds to the radius of the additional area that becomes available to the location of the conidium by the expansion of the alveolus.

### Active migration of alveolar macrophages

AM migrate on the surface of the AEC inside the alveolus and can enter or exit it via the entrance ring or the pores of Kohn. In the agent-based model, this is realized by migration vectors that connect positions on the inner surface of the three-quarter sphere. Migration vectors represent arc-elements to meet the bound-to-surface condition and have a geodesic length of 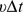, where 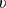 is the specified speed of the AM and 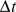 is the timestep. A migration vector 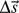 from a given position 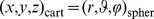 on the surface of the sphere is computed by
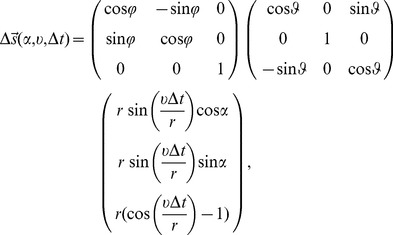
(3)where 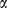 is the direction angle, comparable to the polar angle in a two-dimensional polar coordinate system.

We implemented two migration modes for AM: (i) persistent random walk (PRW) and (ii) biased persistent random walk (BPRW). The PRW is realized by randomly choosing a new direction 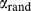 from a uniform distribution over 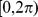 after expiration of the persistence time *t*_P_. In each timestep 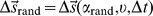 is computed to account for the curvature of the alveolus. We implemented this migration model for constant speed 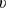 and constant persistence time *t*_P_.

The BPRW assumes that the migration of AM is not purely random but is biased by additional signals. This means that the cell follows the combination of the random migration vector 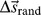 and the signal migration vector 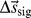 that will be described in the next paragraph. The relative contributions of the two vectors depend on the geodesic distance of the AM from the signal source.

### Chemotactic signalling by alveolar epithelial cells

**Figure 2:**
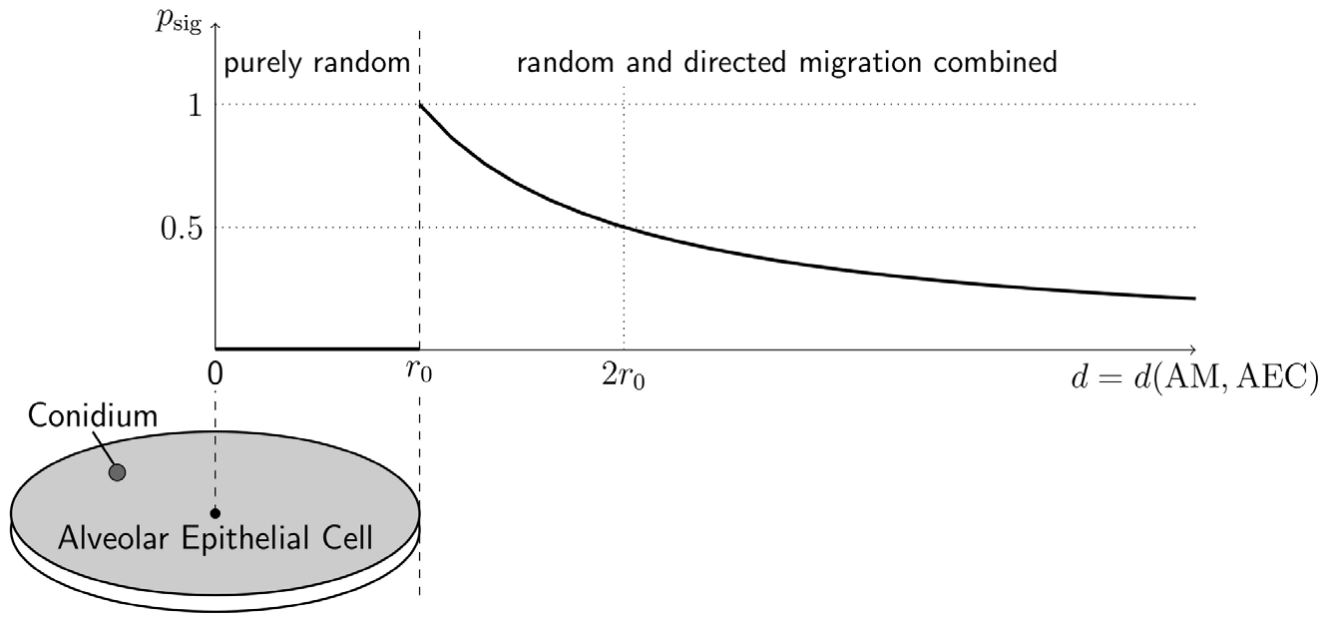
Chemotaxis model for signalling alveolar epithelial cells. Probability of directed migration by AM towards the AEC associated with the conidium as a function of geodesic distance *d* between the centroids of the AEC and the AM. See text for details.

The signal that induces the BPRW is derived from the AEC being in close contact with the conidium. We assume chemokine secretion from either type I or type II AEC in order to direct AM to itself as shown in Fig. 2. The distribution of the secreted molecules follows a two-dimensional diffusion equation with constant source term. We use the steady state solution for the planar case (see Text S1), where the concentration gradient only depends on the distance 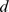 from the source and is proportional to 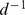. AM migrate with probability 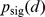 in the direction of the gradient until they reach the AEC associated with the conidum:
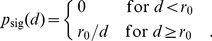
(4)Here, 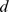 is the geodesic distance from the source of the signal to the centroid of the AM and 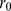 defines the radius of a circular shape with the centroid of the occupied AEC as origin. For our studies we set 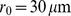 and 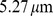, respectively, for the typical radii of AEC of type I and type II. The resulting migration vector for BPRW is computed as follows: First, we apply the relative contributions of random migration
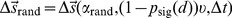
(5)and directed migration
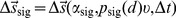
(6)in random order. Since the resulting shift of their linear combination has not necessarily a geodesic length of 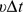, we scale to the geodesic length that corresponds to speed 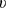. As in the case of PRW, the direction angles 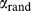 and 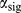 are updated after the persistence time 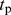 has elapsed. By using the function 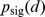 we set a vanishing gradient on the AEC associated with the conidium, assuming a fairly homogeneous chemokine-concentration on the AEC leading to a random migration within the distance 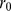. Outside this circular area of radius 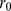, the strength of the gradient is used as a measure for the impact of directed migration.

### Distribution of cells in the alveolus

Assuming that 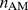 AM are distributed randomly over the 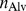 alveoli in the human lung, a 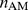-ary Bernoulli experiment with 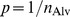 leads to a Binomial distribution
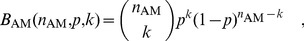
(7)denoting the probability of having 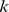 AM per alveolus. For the 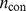 *A. fumigatus* conidia we derive with the same approach the probability 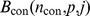 for 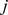 conidia per alveolus. In Table 3 we present the values for parameters used in formula 7.

**Table 3:** Model parameters for human alveolar macrophages.

parameter	description	value	comments
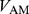	volume	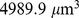	[39]
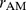	radius	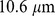	[39]
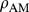	surface density	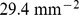	[40]
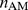	overall number	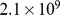	[40], estimation

### Dynamics and boundary conditions

The alveolus consists of two different types of boundaries, where AM can enter and exit: the alveolar entrance ring and the pores of Kohn. We implemented absorbing boundaries with a constant input rate 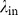 in order to reproduce the Binomial distribution of AM at any time 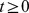 *in silico*. A migrating AM is removed from the virtual alveolus as soon as its centroid has crossed one of the boundary segments. For each parameter combination of AM speed and persistence time the input parameter 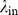 is calibrated to the corresponding Binomial distribution of AM per alveolus (see Text S1). An AM enters the alveolus after the exponentially distributed waiting time
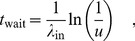
(8)is elapsed. Here, 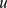 is a uniformly distributed variate in 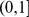. Note that we always draw a new waiting time after the previous waiting time is elapsed. The position of AM insertion into the system follows a uniform random distribution over the length of the line elements belonging to all boundaries. If the position at the boundary is occupied by either an AM or the conidium, we reject that position and choose a new one until a non-occupied boundary position is found.

### Virtual infection scenario and first-passage-time measurements

We vary the corresponding parameter sets in typical ranges in order to analyze the resulting FPT. The Binomial distribution for the number of conidia per alveolus gives evidence for only one conidium in one alveolus as by far the most probable situation in non-empty alveoli. The alveolus is constructed by randomly placing the AEC and the pores of Kohn as explained in the paragraph *Setup of the alveolus*. We start simulating at time *t*  =  0, at which the conidium has entered the alveolus. The number of initial AM is chosen according to the Binomial distribution of AM per alveolus. These AM are placed randomly over the entire surface of the three-quarter sphere. The system dynamics is simulated over time until the first physical cell-cell contact is detected between one of the AM and the conidium of *A. fumigatus*. This is the event of first passage and the time associated with it is referred to as first-passage-time (FPT). The whole scenario is repeated 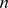 times for each parameter configuration to account for the stochasticity in the computer simulations of the agent-based model (see Table 2). From the distribution of FPT for a given parameter configuration, we calculate the mean, the standard deviation and the median. The results are compared with the typical time of six hours required for *A. fumigatus* conidia to germinate [23]. In the unlikely event that a conidium exits the alveolus due to passive movement in the presence of breathing, the simulation is discarded and a new one is initiated.

### Algorithm and implementation

**Figure 3:**
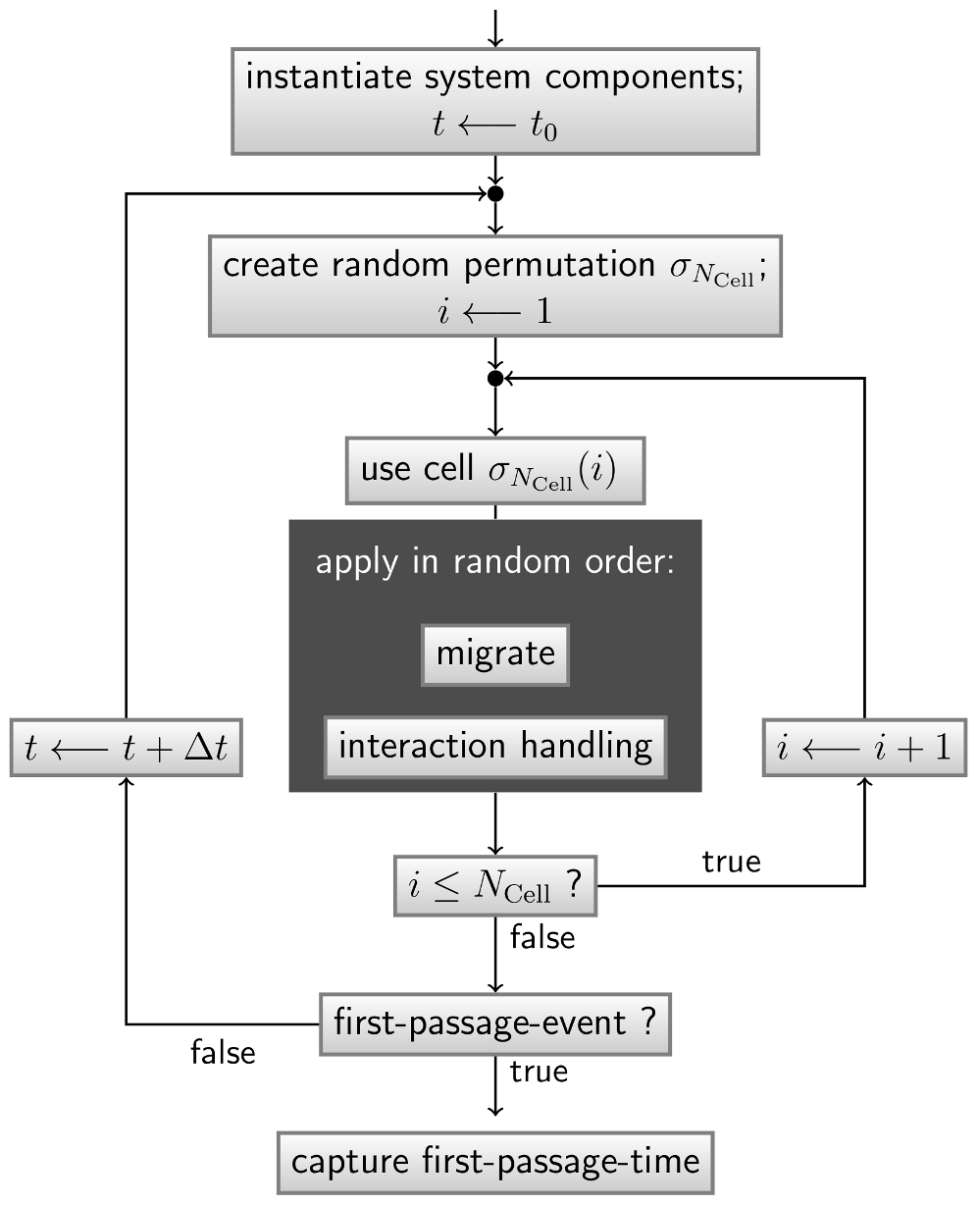
Flowchart of the agent-based simulation procedure for first-passage-time measurements. Integration of the system dynamics over time with timestep 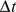 by using an asynchronous random order updating scheme [24]. Here, the recording of one first-passage-time sample is shown. 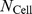 is the total number of cells in the system at time 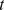 and 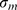 denotes a uniform random permutation of *m* elements. See text for further details.

A general object-oriented framework for agent-based simulations is implemented in the programming language C++. We apply an asynchronous random order updating scheme to integrate the dynamics of the system over time [24]. Here, for each timestep a uniform random permutation 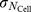 of all cells present in the system at time 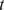 (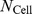) is generated. The flowchart of the agent-based simulation framework is presented in Fig. 3.

The handling of interactions is divided into two parts: First, the currently enabled interactions have to be detected and, second, they are executed. Two dynamic cells interact if their associated spheres overlap or touch each other. Here, we implement a mechanism that avoids overlaps between the spheres of the cells by shifting the most recently moved cell back to a position where it only touches its interaction partner. The simulation ends in the case of an AM touching a conidium for the first time.

Simulations are carried out on a SUSE Linux Enterprise Server version 11 on a x86-64 architecture with 512 GB RAM and 48 AMD Opteron processors, each running on 1781 MHz. Run-time measurements are presented in Table S1. We effectively reduce the run-time over 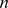 repetitions by executing FPT simulations in parallel.

## Results

### Virtual infection model reveals most probable configuration

**Figure 4:**
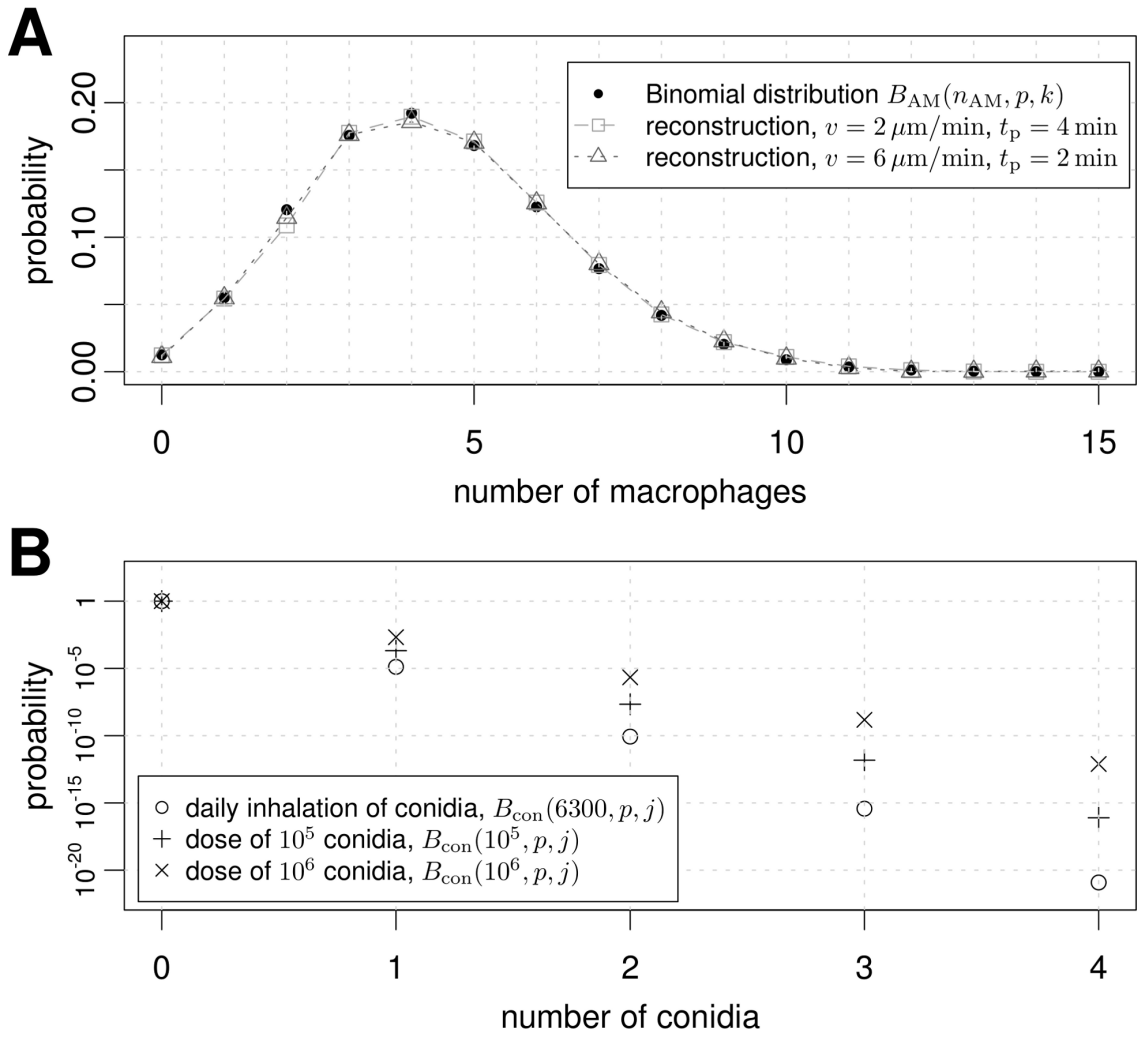
Number of conidia and alveolar macrophages per alveolus. A Binomial distribution is used to get insight into the number of cells per alveolus. (A) The probability distribution of AM per alveolus derived from their overall number (see Table 3) is shown in blue. The quality of the calibration can be seen by means of two example simulations of the persistent random walk migration mode with breathing being disabled. (B) Probability for the distribution of *A. fumigatus* in one alveolus is plotted on a logarithmic scale. Different typical doses as used in experiments are plotted for comparison. Daily inhalation refers to a dose of 6300 conidia, which is taken as the upper threshold of the lungs' daily exposure toward *A. fumigatus*.

The *in silico* experiments were performed for a three-quarter spherical alveolus corresponding to the configuration with highest frequency of occurrence in humans [18]. Alveolar macrophages (AM) were positioned in the alveolus and could migrate on the inner surface and enter or exit the alveolus via the alveolar entrance ring and the pores of Kohn (see Fig. 1 and Video S1). The Binomial distribution of AM in the alveolus was obtained as the result of balancing stochastic AM exiting from the alveolus by fitting a constant input rate 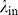 of AM entering the alveolus (Table S2). This input rate was calibrated for each set of migration parameters, *i.e.* AM speed and persistence time, but were found to be fairly independent of the applied migration mode and breathing scenario (see Figs. 4A and S1).

The resulting fits to the Binomial distribution are presented in Fig. 4A for two different sets of migration patterns. We found that each alveolus is on average occupied by 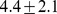 AM over time. As can be seen in Fig. 4B by far the most of all alveoli do not contain a conidium, irrespective of the inhaled dose within the tested range of 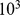 to 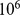 conidia. Of all alveoli that do contain conidia, the configuration with one single conidium is by far the most probable one and it is more than three orders of magnitude less probable to find an alveolus with two or more conidia. The most probable configuration as revealed by the virtual infection model, therefore, corresponds to an alveolus with one conidium and a dynamically varying number of about two to seven AM. In other words, the relevant physiological scenario that we will focus on in the following resembles the problem of finding the needle in the haystack within a time limit that is set by the germination time of about six hours for *A. fumigatus* conidia.

### Randomly migrating AM do not find fungal conidia before germination

Since it is not possible to observe AM migration *in vivo* with today's imaging techniques, nothing is known about their migration behaviour under physiological conditions. However, the virtual infection model allows investigating different hypotheses for AM migration. Here, we started with the hypothesis that AM perform a persistent random walk (PRW) and we scanned the parameter space, which is spanned by the constant speed and persistence time, within reasonable ranges of the parameter values.

A simulation was performed starting from a random initial configuration, *i.e.* the spatial arrangement of alveolar epithelial cells (AEC) forming the alveolus and of the pores of Kohn were randomly chosen within the three-quarter spherical alveolus. Furthermore, the initial condition with regard to the number and position of AM as well as the position of the conidium were also randomly set. We computed the first-passage-time (FPT), *i.e.* the minimal time it takes an arbitrary AM to find the conidium in the alveolus, and repeated this simulation for 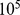 random realizations (Video S2).

**Figure 5:**
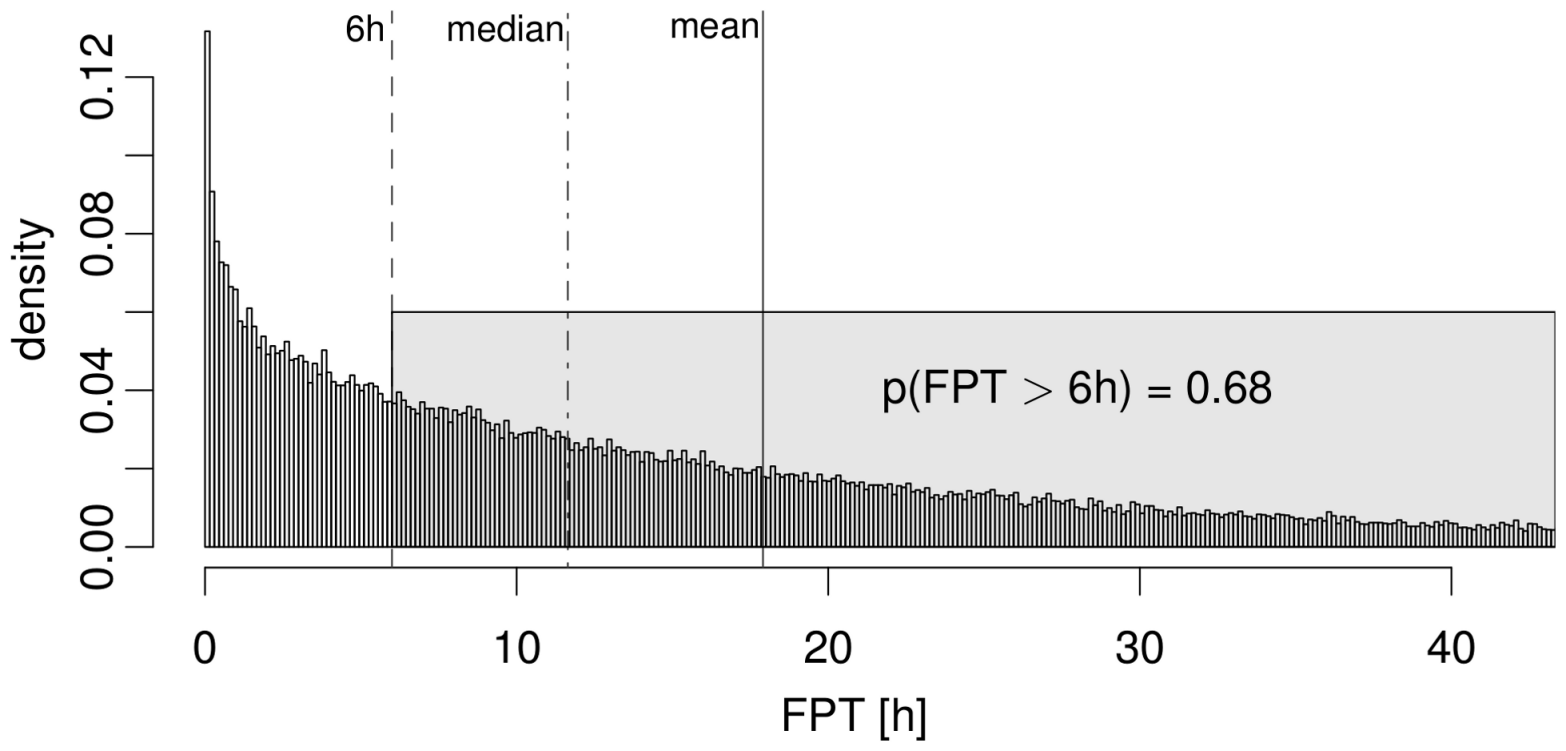
Typical measures of the first-passage-time density distribution used in this study. Here, we show an example-distribution for a persistent random walk scenario with the parameters 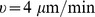 and 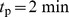 based on 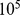 samples (the corresponding distribution for biased persistent random walk is shown in Fig. S2). One sample of these simulations can be viewed in Video S2.

In Fig. 5, we show the result of the FPT distribution for a PRW with parameter values speed 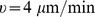 and persistence time 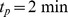. Of note, these distributions typically show long tails, such that despite of the peak value with a FPT well below six hours the probability to find the conidium at more than six hours post infection, 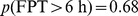, is relatively high. In fact, the median and mean of this distribution was found to be 11.5 hours and 17.5 hours, respectively, thus, reaching inacceptable high values of the order of one day.

**Figure 6:**
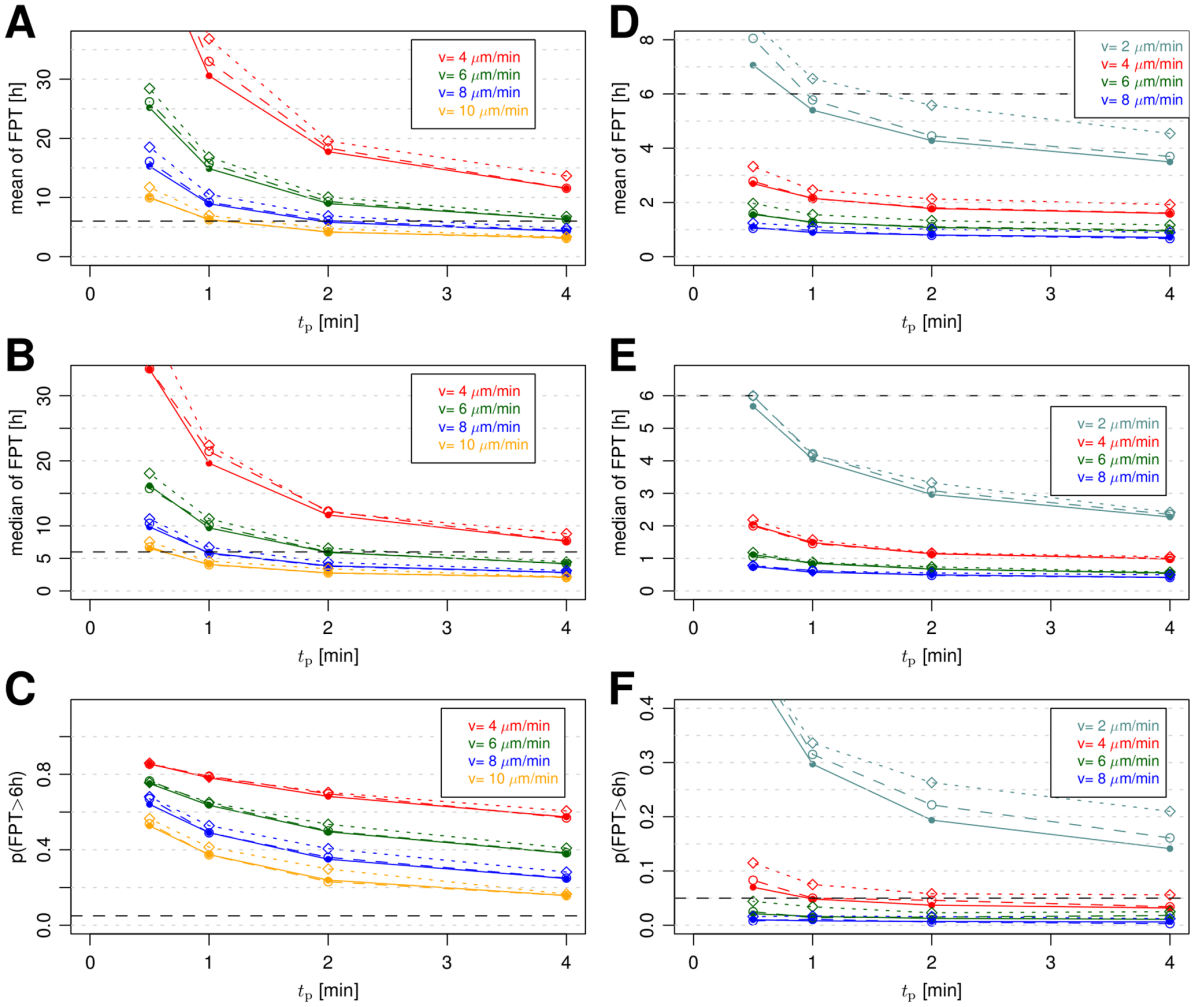
Comparison of first-passage-time distribution measures with and without breathing for both migration modes. Subfigures (A)–(C) show the FPT measures mean, median and probabilities of FPT above six hours for persistent random walk migration mode. Subfigures (D)–(F) show these measures for biased persistent random walk. The two breathing scenarios, resting condition (dashed lines) and heavy exercise (dotted lines) are shown together with the static case (lines) of a constant deflated alveolus. The parameters for the static case and the breathing scenarios are summarized in Table 2.

The results of a systematic investigation of the parameter space for the migration mode PRW are presented in Fig. 6A–C in terms of the mean, the median and the probability 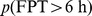. Here, we scanned the parameter values in the range 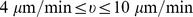 and 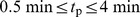. It is observed that the mean FPT and the median FPT decrease with increasing speed and/or persistence time. For example, for a speed of 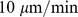 and a persistence time of 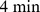, the mean FPT reaches values below three hours. However, even for these extremely high values, the probability that the conidium is not found within six hours post infection remains at relatively high values of 15%.

In addition to studying static alveoli, we also investigated two breathing conditions – referred to as resting and heavy exercise – that differ in the breathing frequency and in the dynamic change of the alveolar volume (see Table 1 and Video S3). As explained in detail in the Model section, breathing conditions are associated with the passive random movement of the *A. fumigatus* conidium due to expansion of the inner surface of the alveolus. The results are shown in Fig. 6A–C by the dashed (resting condition) and dotted (heavy exercise) lines. It is observed that, while the overall change compared with the static alveolus is small, the induced random migration of the conidium generally has a prolonging effect on the mean and median of the FPT as well as on increasing effect on the probability 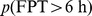. This result can be explained by the fact that the surface area during each cycle of breathing is increased compared with the static case of the deflated state alveolus. Correspondingly, the observed deviations are larger for the heavy exercise condition than for the resting condition. However, the main result of investigating breathing conditions is that their general impact on the FPT is small.

### Virtual infection model predicts chemoattraction of alveolar macrophages

To accomplish the task of finding the needle in a haystack within a time limit, we investigated a second migration mode of AM assuming that they are directed towards the conidium by chemokines released from AEC. More specifically, it is assumed that the AEC associated with the conidium senses the pathogen and in response releases a chemoattractant that diffuses in the surfactant on the inner surface of the alveolus and guides AM the way to the AEC (see Fig. 2). Once a randomly migrating AM detects the chemotactic gradient, it becomes attracted to the respective AEC and arriving at this cell continues with a random search for the conidium on its surface (see Video S4). Since this migration mode is both random and transiently directed (see Eqs. 5 and 6), we refer to it as biased persistent random walk (BPRW).

In Fig. 6D–F, we present the results of FPT measurements for the BPRW. In comparison with the PRW (see Fig. 6A–C), it is observed that the mean and the median of the FPT were significantly reduced by one order of magnitude. Only in the case of very small speed values for AM, e.g. 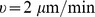, we found for the probability 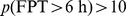
% over the whole range of tested persistence times. In general, this AM migration mode does accomplish the task of finding the conidium reasonably well, because 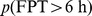 remains well below 5% for all combinations of 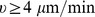 and 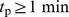. These observations remained again largely unchanged for both breathing conditions (see Fig. 6D–F), which motivated us to concentrate on the static alveolus in what follows.

**Figure 7:**
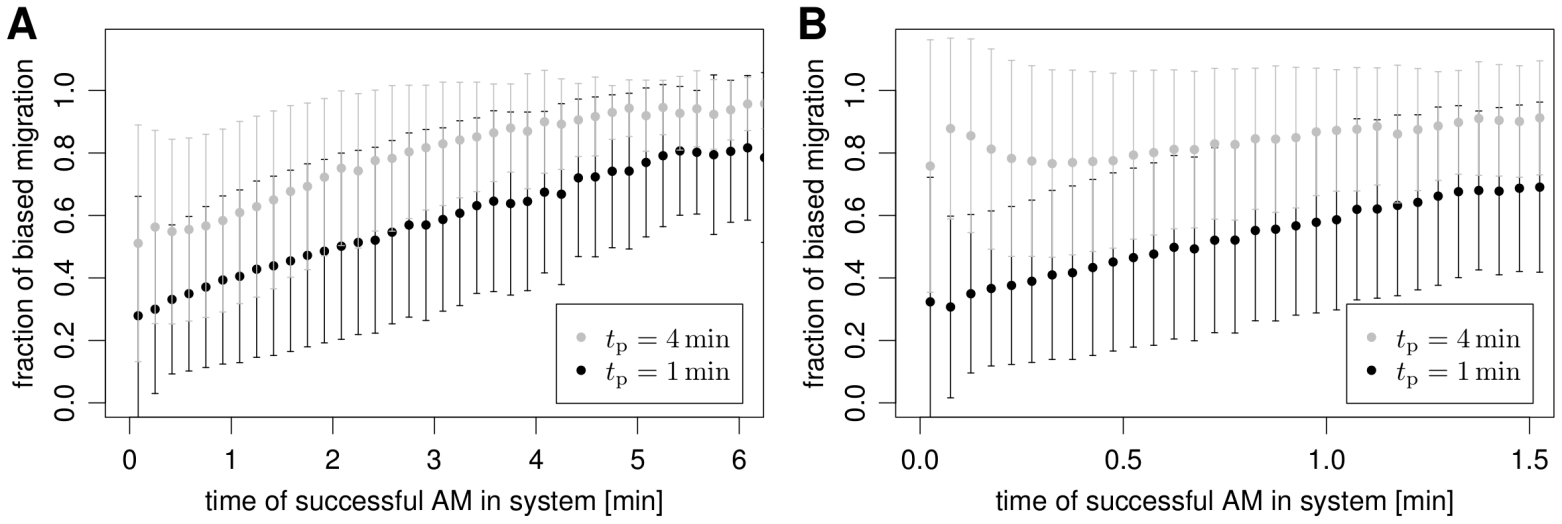
Time fractions for successful AM performing biased persistent random walk migration. The fraction of the biased migration part (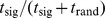, see text for details). (A) Mean and standard deviation of the fraction of biased migration for 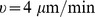, (B) for 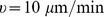. The overall number of samples is 10^5^.

As mentioned above, for this migration mode the FPT consists of two parts: First, randomly migrating AM are biased by the chemotactic signal attracting them towards the AEC associated with the conidium and the duration of time that this process takes was measured by 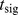. Second, the search for the conidium on the respective AEC obeys a persistent random walk and is associated with the time duration 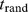. We analyzed the relative contributions of these time durations for different parameter values of speed and persistence time by computing the time fraction of the purely random migration on the AEC, i.e. 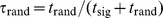, and that of the chemotactic migration towards this AEC: 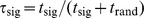. This computation was done in retrospect for the AM that successfully found the conidium. The results for selected speeds of 4 and 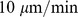 and selected persistence times of 1 and 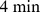 are presented in Fig. 7. In agreement with our earlier observations for PRW migration in the alveolus, we find that higher AM speeds and/or persistence times also reduce 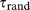 relative to 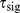, if the random search is restricted to a single AEC. Thus, low speed values and/or persistence times result in the major contribution to the FPT associated with the random search of AM on the AEC.

**Figure 8:**
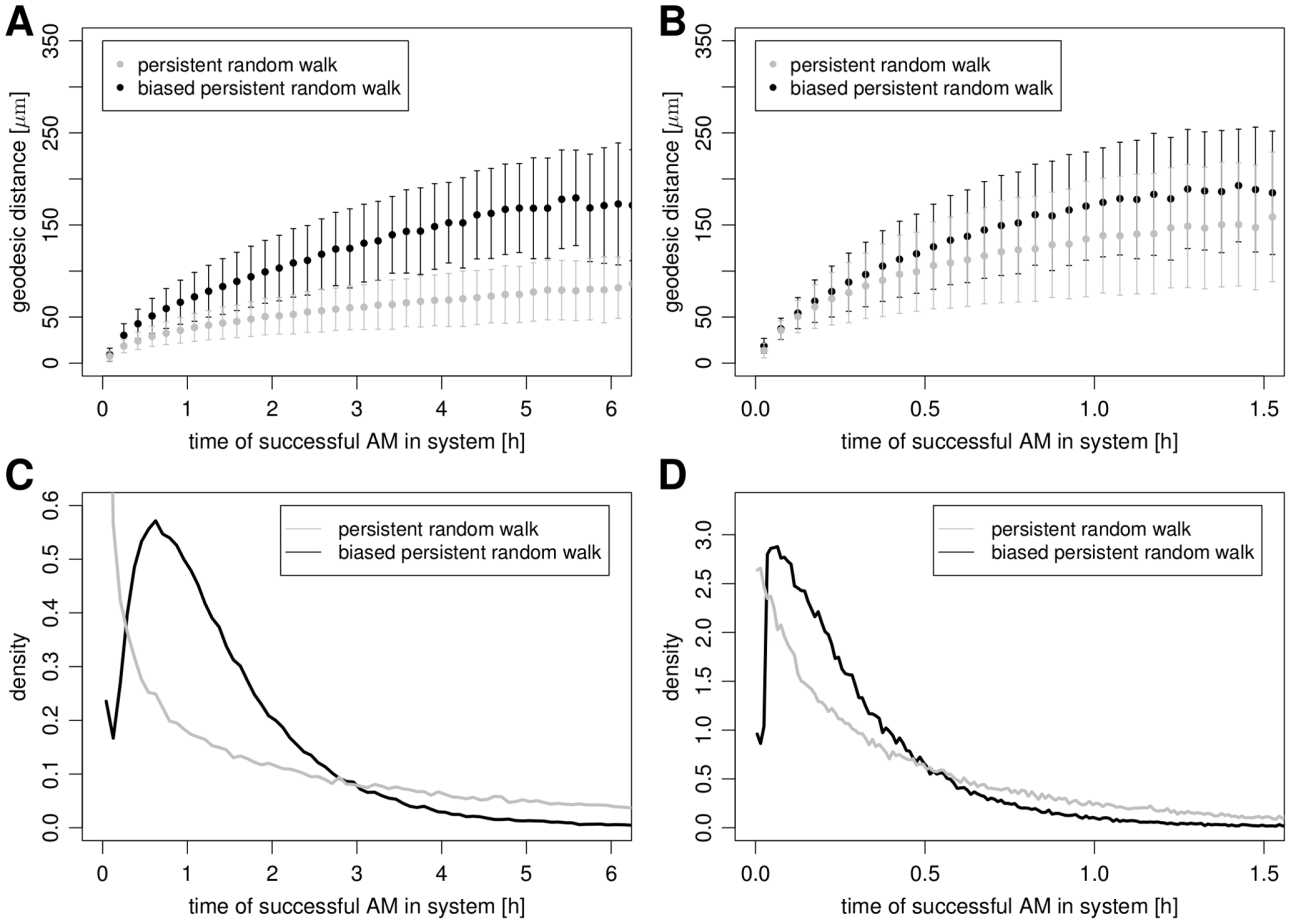
Geodesic distances and the retention time distribution of successful macrophages for different migration modes in the static case. (A) and (C) are based on 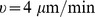 and 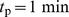, whereas (B) and (D) are based on 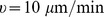 and 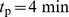.

Next, we computed the distance corresponding to a geodesic in the spherical geometry denoting the shortest distance between the conidium and the successful AM on first occurrence in the system (see Fig. 8A and B). It is observed that for both migration modes the average geodesic distances typically level off at about 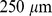, implying that successful AM are closer to the conidium than one third of the circumference of the spherical alveolus. The magnitude of this distance is consistent with theoretical estimates for maximal communication distances between single cells by chemokines [25]. Furthermore, geodesic distances for higher persistence times or higher speeds are larger suggesting advantages for a fast exploration of the spatial environment, thus, having reduced FPT.

### Search strategies of AM disentangled

The virtual infection model allows for the disentanglement of AM search strategies. This is achieved by comparing the two migration modes through an in-retrospect analysis of AM properties that successfully found the conidium. In other words, we investigated what was special about the AM that accomplished the task of finding the needle in a haystack most quickly. The results of this analysis are summarized in Fig. 8, where we computed the retention time distribution, *i.e.* the distribution of times that the successful AM was in the system. Interestingly, we found that this distribution is diverging at short time durations for the PRW resembling an exponential distribution. This is shown in Fig. 8C and D for two different sets of parameter values 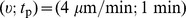 and 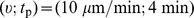. In contrast, in the case of the BPRW this distribution showed a peak at some finite time and this peak time was lower for higher values of speed and/or persistence time. This suggests that in the case of PRW migration, successful AM were mostly those that happened to enter the alveolus at entry points close to the position of the conidium and could find them right away. Inspecting again the geodesic distances – being the shortest distance between the successful AM on first occurrence in the system and the conidium – confirms this interpretation. As can be seen in Fig. 8A and B, the distance of the successful AM that performed PRW migration is vanishing, whereas for BPRW migration this distance remains finite. The average geodesic distance of the successful AM from the conidium for 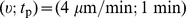 was found to be 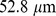 for the PRW and attained a higher value of 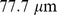 for the BPRW (see Fig. 8A). Similarly, for 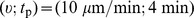 we found an average geodesic distance of 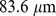 for the PRW and 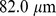 for the BPRW (see Fig. 8B).

These observations were further investigated by averaging over the mean values extracted for each of the 20 parameter combinations, *i.e.* 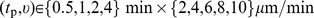. This revealed the geodesic distance of the successful AM from the conidium to be 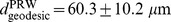 for the PRW and to attain the significantly higher value of 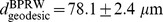 for the BPRW. Thus, the successful AM performing PRW start closer to the conidium than is the case for the BPRW, but this proximity can only be realized by random entries into the alveolus (via the alveolar entrance ring or the numerous pores of Kohn). Since it is statistically more likely that AM enter the system within the area defined by the larger distance, 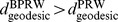, the successful AM of the BPRW will find the conidium earlier than the successful AM of the PRW. This also explains why the FPT of AM that are migrating by a PRW is significantly longer: they do not find their target by capability but mostly by the luck of a fortunate initial condition or of entering the alveolus at an entry point being close to the conidium. In contrast, for BPRW migration the impact of luckily entering the alveolus close to the conidium is less important and AM find their target more by the capability of following the chemotactic signal.

## Discussion

In this study, we investigated the early immune response to *A. fumigatus* infection involving the recognition of conidia by alveolar macrophages (AM). Computer simulations of a realistic agent-based model for the human alveolus predicted that randomly migrating AM will not find a conidium before germination. The results provided strong evidence that chemotactic signals released by the alveolar epithelial cell (AEC) associated with the conidium are required to comply with the widely accepted role of AM in the detection and recognition of conidia during the early immune response [10], [26].

Due to the peculiar physiology of the human lung and the small number of conidia relative to the number of alveoli, the infection scenario is not directly observable *in vivo* under realistic conditions. Therefore, computer simulations of the agent-based model providing the distribution of first-passage-time (FPT) as simulation readout of the virtual infection scenario for varying parameters of different AM migration modes represent a most appropriate approach for this feasibility study. The human alveolus was implemented in the agent-based model as a three-quarter sphere, *i.e.* corresponding to the most common alveolar structure [18], with type I and type II AEC as well as pores of Kohn of realistic morphologies and population numbers [19]. As the number of interacting cells involved in the simulations is low, the model may appear simple on first sight; however, the complexity of the model is associated with the system's open boundaries. Since AM can only exit the alveolus by approaching the entrance ring or the randomly positioned pores of Kohn, balancing the equilibrium distribution is a critical issue in this dynamic system and was solved by the concept of waiting times for entering AM. To the best of our knowledge this is the first agent-based model of a human alveolus in the presence of respiration.

At first, we computed the FPT distribution for a persistent random walk as migration mode of AM. For reasonable AM speed values of 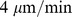, we obtained relatively high FPT values of 10–48 hours on average for persistence times in the range of 0.5–4 minutes. This result has two possible implications: (i) the role of AM in *A. fumigatus* infection, which recently was controversially discussed [13], [26], may be overestimated or (ii) the persistent random walk (PRW) hypothesis may not be valid for AM migration. Therefore, in a second step, we investigated the possibility that AM perform a biased persistent random walk, *i.e.* AM migration is not purely random but guided by chemotaxis towards the AEC that is associated with the conidium. In this case, our computer simulations revealed a significant drop in the FPT of about one order of magnitude. In particular, for reasonable AM speeds starting from 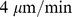 the conidium was detected within six hours post infection in more than 95% of all cases. It should be noted that these results were obtained for a conservative implementation of chemotaxis, where AM migration was composed of random and directed contributions to the AEC but not directly to the conidium on the AEC itself. Thus, the current implementation assumed that, once AM arrived at the AEC associated with the conidium, the conidium itself had to be searched randomly on this AEC. Furthermore, a geodesic distance analysis showed that successful AM typically catched the chemotactic signal within a geodesic distance of at most 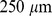, which is in good agreement with theoretical estimations of maximal cell-to-cell communication distances [25].

Furthermore, we considered the possibility that the onset of chemotaxis is retarded because swelling of conidia associated with the exposure of their surface molecules might be required for AEC to initiate chemokine release. If chemotactic signalling is initiated by swollen conidia only, a time lag of about three hours post infection applies [23]. Nevertheless, our simulations of the BPRW revealed that conidium detection within six hours post infection was maintained in 95% of the cases, as long as AM attained speed values of 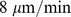 or higher (see Fig. S3 in comparison with Fig. 6F).

Recently, evidence is emerging that neutrophils rather than AM may be the predominant phagocytes to detect and clear *A. fumigatus* infection [13]. In contrast to our study, millions of conidia were administered to each mouse, which is far from normal conditions for humans that inhale between four and five conidia per minute. As a consequence of providing a superdose of conidia, strong and immediate inflammation, e.g. induction of neutrophil recruitment, can be expected. In this context, the question arises how the recruitment of neutrophils is regulated by the inflammatory response. It is well-known that neutrophils can be attracted by activated AM trough the secretion of IL-8 [6]. However, while this would imply that detection of conidia by AM precedes, one could as well imagine that neutrophils are recruited by other signals, e.g. by chemokines released from AEC of type II directly [27]. In any case, our computer simulations revealed that a chemotactic signal would as well be required for neutrophils, because PRW requires speed values of phagocytes well above 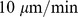 for timely detection of the conidium to achieve a success probability of at least 95%.

Another option for the realization of pathogen detection arises from a recent *in situ* setup, which suggests that sessile AM located at certain spots in the alveoli detect pathogens that are washed towards these phagocytes [28]. However, this process was not quantified and it remains unclear to what extent AM migration activity is directly affected in this *in situ* study. For the sake of completeness, we scanned the whole range of measured speed values for AM. Furthermore, the fluidic surfactant lining layer could also contribute to the recruitment of neutrophils to the site of infection by pro-inflammatory- and chemoattracting anaphylatoxin and C3a molecules that are cleaved from C3 molecules on the fungal surface [29], [30]. However, the impact of complement molecules on the early inflammatory response against *A. fumigatus* is currently unknown [31]. Note that AEC may also play a part in this regard, since these are in direct contact with the conidia from the time of their entry. AEC of type II were shown to secrete IL-8 on contact with swollen conidia of *A. fumigatus* in a TLR-independent fashion [27] and are able to uptake condia within two hours of co-incubation *in vitro* [32]. Due to the fact that type I AEC are diffcult to isolate, immune-regulatory functions were so far exclusively investigated for AEC of type II. It should be noted that type I cells comprise 95% of the alveolar surface [19], which means that a vast majority of conidia entering the alveoli will most certainly make contact with type I AEC.

Search-strategies of AM in the human alveolus are of general interest, because apart from *A. fumigatus* several other harmful fungal spores as well as pathogenic bacteria may cause damage or lead to infectious diseases. In the present study, we focused on *A. fumigatus* because this pathogen sets a tight time scale for removal by phagocytes before germination and invasion of AEC. AM own the capability of recognition and phagocytosis of resting conidia [26], as rigorously quantified by automated image analysis [33], but to figure out the exact mechanisms that lie behind the process of seeking the fungal intruder, we suggest an experimental setup that allows distinguishing the interactions of the different AEC types with AM in the presence of various pathogens. For example, using transwell assays, the hypothesis of directed AM migration due to chemotactic signalling by AEC may be experimentally investigated [34]. Furthermore, if *in vivo* imaging of alveolar ducts could be performed under physiological conditions, typical migration modes in the presence and absence of *A. fumigatus* conidia may be revealed by automated characterization and parameter-free classification of cell tracks [35]. Based on such data, the parameters of the agent-based model could be refined and initiate the iterative cycle between experiment and theory within an image-based systems biology approach.

## Supporting Information

Figure S1 ***In silico*** **reconstruction of the Binomial distribution for AM under different breathing conditions and migration modes.**
(TIF)

Figure S2
**Typical first-passage-time density distribution for biased persistent random walk.** Biased persistent random walk migration of alveolar macrophages with parameters 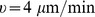 and 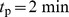 based on 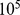 samples.(TIF)

Figure S3 **Probability of FPT being above three hours for biased persistent random walk migration.** The reduction of the maximal FPT from six to three hours mimics the case where fungal swelling is required for type I AEC to release chemokines.
(TIF)

Table S1
**Average run-time for one timestep** 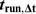 **of agent-based simulations for different breathing conditions and migration modes.** Mean run-times and their corresponding standard deviation are averaged over 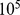 runs in the static case and averaged over 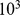 runs under resting and heavy exercise breathing conditions.(PDF)

Table S2
**Calibrated input rates** 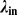 **for the different migration mode parameter configurations of AM.**
(PDF)

Text S1 **Construction of the alveolus and its composition, estimation of cell numbers, diffusion of chemokines and run-time profiling of the simulations.**
(PDF)

Video S1 **Reconstruction of the spherical three-quarter alveolus.** Sizes, shapes and cell numbers are summarized in the Table 1 and in the Model section of the main article. AEC of type I (yellow), AEC of type II (blue) and the pores of Kohn (black) are visible in the morphology of the three-quarter alveolus.
(MP4)

Video S2
**Virtual infection scenario with persistent random walk migration of alveolar macrophages.** Alveolar macrophages (green) perform persistent random walk migration with parameters 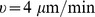 and 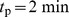 in order to find the conidium of *A. fumigatus* (red) in the alveolus. In a time period of 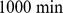 the alveolar macrophages did not complete the first-passage. The video is sampled at a rate of 25 frames per second and the time between two frames corresponds to 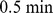 real time.(MP4)

Video S3
**Alveolar macrophages in an alveolus with resting breathing condition.** Inflation and deflation of the three-quarter alveolus are shown in real time. In the resting breathing condition, we apply a respiration frequency of 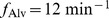. A fixed cubic bounding box around the alveolus is used as static reference.(MP4)

Video S4
**Virtual infection scenario with biased persistent random walk migration of alveolar macrophages.** Alveolar macrophages (green) perform biased persistent random walk migration with parameters 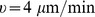 and 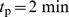 in order to find the conidium of *A. fumigatus* (red) in the alveolus. The alveolar macrophages realized the first-passage in 90 min of time. The video is sampled at a rate of 25 frames per second and the time between two frames corresponds to 0.5 min real time.(MP4)
