# Supplementary figures and images for: Agent-Based Model of Human Alveoli Predicts Chemotactic Signaling by Epithelial Cells during Early Aspergillus fumigatus Infection
Source: PLoS One. 2014 Oct 31;9(10):e111630. doi: 10.1371/journal.pone.0111630 (PMC4216106; doi:10.1371/journal.pone.0111630)

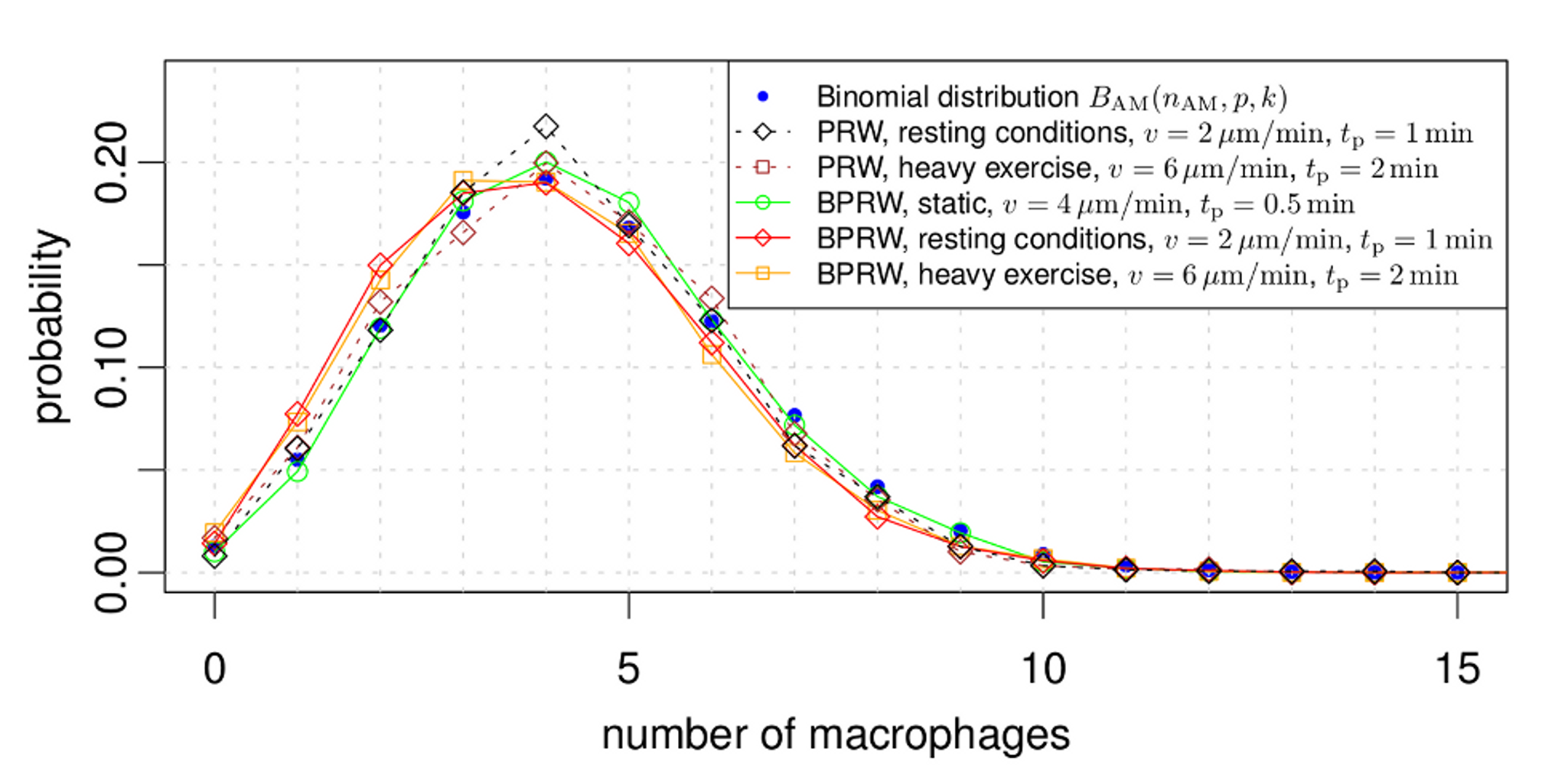

Supplement: Figure S1 — In silico reconstruction of the Binomial distribution for AM under different breathing conditions and migration modes. (TIF) [file pone.0111630.s001.tif]

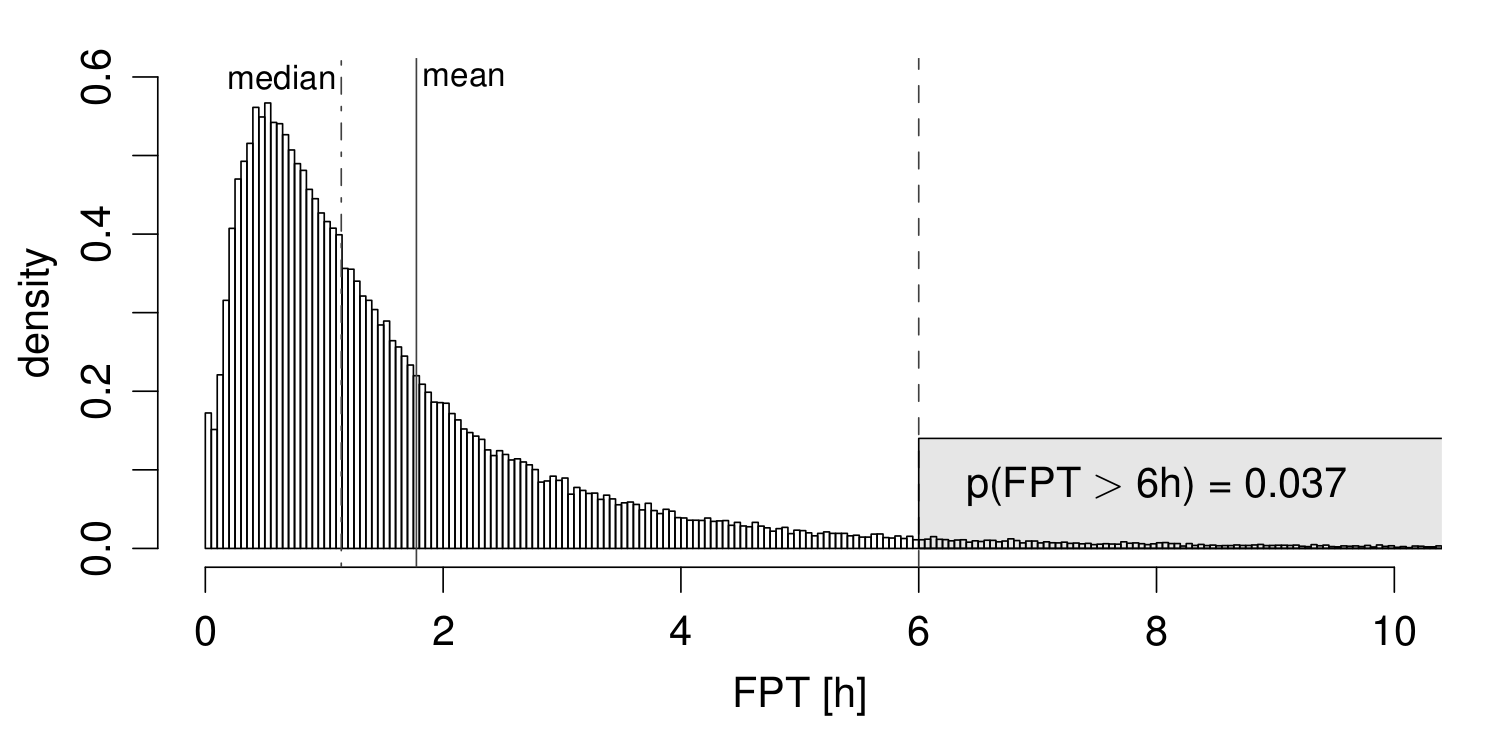

Supplement: Figure S2 — Typical first-passage-time density distribution for biased persistent random walk. Biased persistent random walk migration of alveolar macrophages with parameters and based on samples. (TIF) [file pone.0111630.s002.tif]

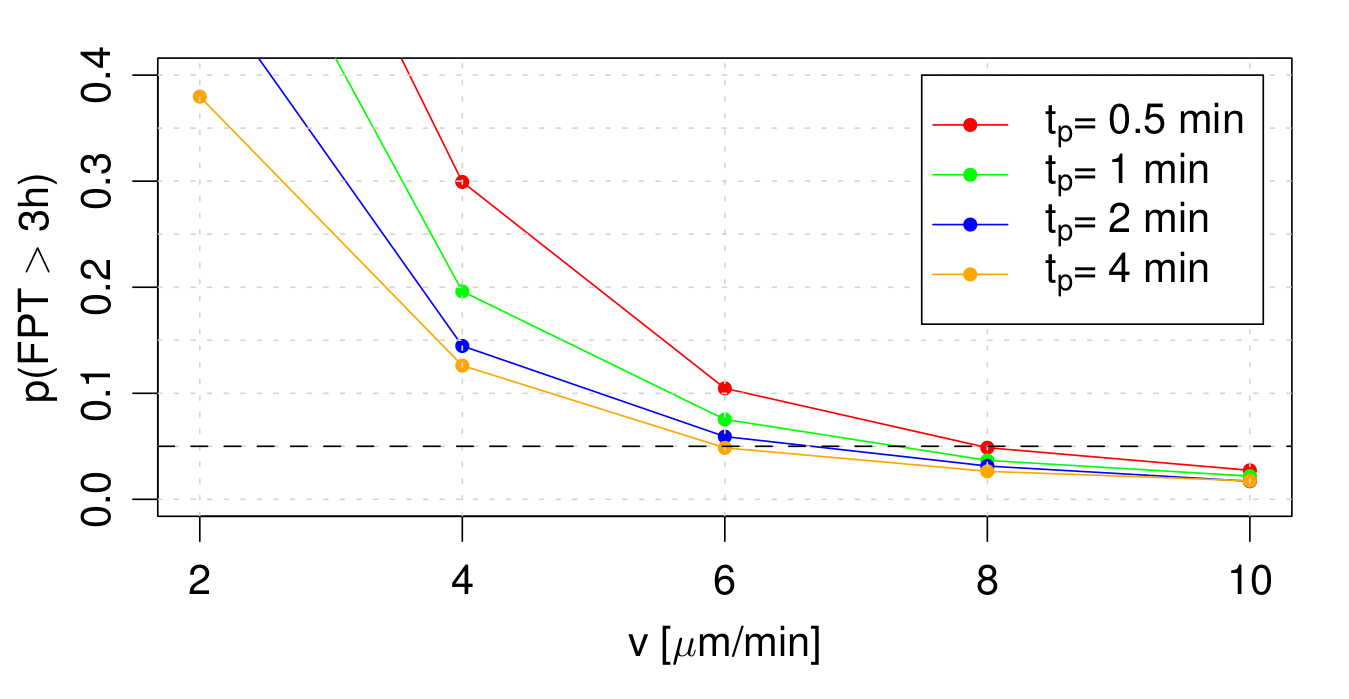

Supplement: Figure S3 — Probability of FPT being above three hours for biased persistent random walk migration. The reduction of the maximal FPT from six to three hours mimics the case where fungal swelling is required for type I AEC to release chemokines. (TIF) [file pone.0111630.s003.tif]
