# Supplementary material for: Agent-Based Model of Human Alveoli Predicts Chemotactic Signaling by Epithelial Cells during Early Aspergillus fumigatus Infection
Source: PLoS One. 2014 Oct 31;9(10):e111630. doi: 10.1371/journal.pone.0111630 (PMC4216106; doi:10.1371/journal.pone.0111630)
Supplement: Table S1 — Average run-time for one timestep of agent-based simulations for different breathing conditions and migration modes. Mean run-times and their corresponding standard deviation are averaged over runs in the static case and averaged over runs under resting and heavy exercise breathing conditions. (PDF) [file pone.0111630.s004.pdf]

Table S1. Average run-time for one timestep $t_{\mathrm{run},\Delta\mathrm{t}}$ of agent-based simulations for different breathing conditions and migration modes. Mean run-times and their corresponding standard deviation are averaged over 10^5 runs in the static case and averaged over 10^3 runs under resting and heavy exercise breathing conditions.

	breathing condition		
$t_{\mathrm{run},\Delta\mathrm{t}}$	static case	resting condition	heavy exercise
Δt	0.1 min	0.001 min	0.001 min
persistent random walk	(0.217 ± 0.075) ms	(0.210 ± 0.074) ms	(0.209 ± 0.069) ms
biased persistent random walk	(0.238 ± 0.109) ms	(0.224 ± 0.099) ms	(0.222 ± 0.098) ms
