# Supplementary material for: Agent-Based Model of Human Alveoli Predicts Chemotactic Signaling by Epithelial Cells during Early Aspergillus fumigatus Infection
Source: PLoS One. 2014 Oct 31;9(10):e111630. doi: 10.1371/journal.pone.0111630 (PMC4216106; doi:10.1371/journal.pone.0111630)
Supplement: Table S2 — Calibrated input rates for the different migration mode parameter configurations of AM. (PDF) [file pone.0111630.s005.pdf]

Table S2. Calibrated input rates λ_{in} for the different migration mode parameter configurations of AM.

	λ_{in} [min^{-1}]	v [μm/min] 2	4	6	8	10
t_{p} [min]	0.5	0.0056	0.01125	0.01675	0.021875	0.0275
	1	0.005725	0.01125	0.01675	0.0225	0.0275
	2	0.005725	0.0115	0.01725	0.0225	0.028
	4	0.006	0.0115	0.01725	0.0225	0.028
