# Supplementary material for: Agent-Based Model of Human Alveoli Predicts Chemotactic Signaling by Epithelial Cells during Early Aspergillus fumigatus Infection
Source: PLoS One. 2014 Oct 31;9(10):e111630. doi: 10.1371/journal.pone.0111630 (PMC4216106; doi:10.1371/journal.pone.0111630)
Supplement: Text S1 — Construction of the alveolus and its composition, estimation of cell numbers, diffusion of chemokines and run-time profiling of the simulations. (PDF) [file pone.0111630.s006.pdf]

Text S1: Construction of the alveolus and its composition, estimation of cell numbers, diffusion of chemokines and run-time profiling of the simulations.

Construction of the Alveolus and its Composition

The most frequent shape of alveoli in humans was found to be the three-quarter alveolus [1]. In order to construct the spherical shape according to the volume fraction, we use a lower threshold for ϑ_c of the polar angle $\vartheta_\mathrm{c} \leq \vartheta \leq \pi$. ϑ_c is computed with the help of the following equation:

$$\pi r^3 = \int \mathrm{dV} = \int_0^{2\pi} \int_{\vartheta_\mathrm{c}}^{\pi} \int_0^{\mathrm{r}} \mathrm{r}'^2 \sin\vartheta \mathrm{dr}' \mathrm{d}\vartheta \mathrm{d}\varphi \quad , \tag{1}$$

which leads to $\vartheta_\mathrm{c} = \pi/3$. It follows that the epithilial surface of the three-quarter alveolus equals $A_\mathrm{Alv} = 3\pi r^2$.

The total number of alveoli in the human lung was estimated in Ochs *et al.* [2]. Here, a stereological analysis was performed to estimate the overall number of alveoli in the lung of adult humans. This yielded the average number of 4.8×10^8, with all measurements being in the range $[2.74, 7.9] \times 10^8$ alveoli.

The spatial arrangement and dynamics of single alveoli are governed by the influx of air and the respiration frequency. The International Commission on Radiological Protection (ICRP) designed a standard model of the human lung and also displayed standard values for the respiration of human beings at different ages and different states of effort [3]. Breathing frequencies and tidal volumes are the important parameters in this respect. Balashazy *et al.* calculated based on the tidal volume of $V_\mathrm{T} = 750\,\mathrm{ml}$ per breath and from an average alveolar radius of $r_\mathrm{Alv,max} = 125\,\mu\mathrm{m}$ in the resting state, the average minimum alveolar radius $r_\mathrm{Alv,min} = 116.5\,\mu\mathrm{m}$ under the assumption that alveoli resize in the same way during one breathing cycle [4]. Under this assumption and for a tidal volume of $V_\mathrm{T} = 1923\,\mathrm{ml}$ per breath during heavy exercise, the maximum alveolar radius reaches $136.5\,\mu\mathrm{m}$.

For the construction of the epithelium it is necessary to know the numbers and sizes of the alveolar epithelial cells (AEC), which consist predominantly of type I and type II AEC. In Herzog *et al.*, it is described that type I cells cover 95% of the surface area in alveoli and the remaining 5% are covered by type II cells [5]. Type I cells have a flat morphology and are huge in comparison to type II cells, which have a cuboidal shape. The proportions of the number of cells differ strongly from the surface coverage relation between type I and type II cells as a matter of the size-differences of the cells. Herzog

et al. reported that type I cells make up 8% of all cells in the adult human lung, whereas type II cells amount to 15%. Stone *et al.* measured the mean cell-volume of AEC type II of human lungs, which was $V_{\mathrm{AECT2,avg}} = 815\,\mu\mathrm{m}^3$ on average [6]. With this value we are able to estimate the number of cells in one alveolus. The length of one AEC type II edge assuming cubic symmetry is given by

$$a_{\mathrm{AECT2}} = V_{\mathrm{AECT2,avg}}^{1/3} \approx 9.3\,\mu m \tag{2}$$

This leads to a surface coverage of the alveolus (assuming a three-quarter sphere) of $A_{\mathrm{AECT2}} = a_{\mathrm{AECT2}}^2$ per type II cell. Knowing that type II cells cover 5% of the alveolar surface, we are able to determine the number of cells in inflated and deflated alveolar state by

$$n_{\mathrm{AECT2,max}} = \frac{0.05 \times 3\pi r_{\mathrm{Alv,max}}^2}{A_{\mathrm{AECT2}}} \approx 84\ , \tag{3}$$

$$n_{\mathrm{AECT2,min}} = \frac{0.05 \times 3\pi r_{\mathrm{Alv,min}}^2}{A_{\mathrm{AECT2}}} \approx 74\ . \tag{4}$$

This leads to the number of type I cells by the relation of the fraction of both cell types:

$$n_{\mathrm{AECT1,max}} = \frac{8\%}{15\%} n_{\mathrm{AECT2,max}} \approx 45\ , \tag{5}$$

$$n_{\mathrm{AECT1,min}} = \frac{8\%}{15\%} n_{\mathrm{AECT2,min}} \approx 39\ . \tag{6}$$

In the model, the AEC of type I are placed first. To reach the amount of cells calculated for a three-quarter sphere it is necessary to introduce the parameter $n_{\mathrm{AECT1,pgc}}$, which is a natural number that defines how many cells occur along a great circles of the sphere. The centroids of the type I cells are placed equidistantly by geodesic length $(2\pi r_{\mathrm{Alv,max}})/n_{\mathrm{AECT1,pgc}}$ over parallels of the sphere, whereas the parallels themselves are equidistantly distributed over the whole sphere by the same geodesic length in the interval $\vartheta \in [\vartheta_{\mathrm{c}}, \pi]$ starting from $\vartheta_c = \pi/3$. The amount of centroids of AEC of type I varies per parallel depending on the fraction of its length to the length of a great circle. The amount of cells per parallel equals $n_{\mathrm{AECT1,pgc}} \sin\vartheta$, where ϑ is the polar angle of the parallel under consideration. Note that parameter $n_{\mathrm{AECT1,pgc}}$ determines the total number of type I AEC n_{AECT1} per three-quarter alveolus. We varied $n_{\mathrm{AECT1,pgc}}$ in order to get a total type I cell number with $n_{\mathrm{AECT1,min}} \leq n_{\mathrm{AECT1}} \leq n_{\mathrm{AECT1,max}}$. We determined a value of $n_{\mathrm{AECT1,pgc}} = 13$ leading to $n_{\mathrm{AECT1}} = 39$ and $n_{\mathrm{AECT2}} = 74$ for the alveolus

model and the simulations. From the centroids of the AEC of type I the three-dimensional Voronoi tesselation is calculated and mapped onto the surface of the sphere. Each Voronoi cell represents the spatial dimensions of one AEC of type I. The pores of Kohn and the AEC of type II are subsequently placed randomly over distinct edges of neighbouring type I cells.

Regarding the pores of Kohn, Kawakami *et al.* performed a study comparing different measures like number per alveolus and axial length of a pore in humans [7]. From an average surface of $11.1 \times 10^3\,\mu\text{m}^2$ for the bottom part of the alveolus and an average of 8.9 pores per bottom part, we are able to estimate the average surface density of the pores of Kohn to be $802\,\text{mm}^{-2}$. From the average fraction of the covered area by pores of Kohn of the bottom part being 0.0225, we obtain a coverage surface of $28.1\,\mu\text{m}^2$ per pore which leads to an average radius of $2.99\,\mu\text{m}$ for a pore assuming circular symmetry of its cross section.

Estimation of Cell Numbers

The density of alveolar macrophages ρ_{AM} is taken from the study of Wallace *et al.*, where smokers and non-smokers were compared [8]. For non-smokers the surface density is $\rho_{\text{AM}} = 29.4\,\text{mm}^{-2}$ on average. Note that the alveoli were in expanded state when they sampled the macrophages in order to compute the density. From the surface density ρ_{AM}, the area covered by one lung alveolus A_{Alv} and the overall number of lung alveoli $n_{\text{Alv}} = 4.8 \times 10^8$, we are able to estimate the total number of alveolar macrophages on average in the following way:

$$\begin{aligned} n_{\text{AM}} &= \rho_{\text{AM}} A_{\text{Alv}} n_{\text{Alv}} = \rho_{\text{AM}} \times 3\pi r^2_{\text{Alv,max}} n_{\text{Alv}} \\ &\approx 2.1 \times 10^9 \,. \end{aligned} \tag{7}$$

Here, we assume that each alveolus has the surface area of a three-quarter sphere in expanded state under resting breathing conditions, which relates to an alveolus radius of $r_{\text{Alv,max}} = 125\,\mu\text{m}$.

From the density of conidia $\rho_{\text{con}} = 194\,\text{m}^{-3}$ [9], we derive the frequencey of spore-inhalation f_{inh} as follows:

$$f_{\text{inh}} = \rho_{\text{con}} V_{\text{T}} f_{\text{Alv}} \quad , \tag{8}$$

where V_{T} is the tidal volume per breath and f_{Alv} the frequency of respiration. Considering resting

breathing conditions, we find $f_{\text{inh}} \approx 6300\,\mathrm{d}^{-1}$. Note that this number relates to the amount of spores that pass into the thorax, but it remains unknown what fraction conidia are able to evade the filter activity of the lung and reach the alveolar ducts.

Diffusion of Chemokines

We apply the two-dimensional planar diffusion equation with a source term:

$$\frac{\partial}{\partial t} c(\vec{d}, t) = D \nabla^2 c(\vec{d}, t) + S(\vec{d}, t) \quad , \tag{9}$$

where $c(\vec{d}, t)$ is the molecule concentration at position $\vec{d} = (x, y)^{\mathrm{T}}$ and time t, ∇ is the Nabla operator and D the diffusion constant. We start from the initial condition $c(\vec{d}, 0) = 0$ and set a constant source term $S(\vec{d}, t) = S_0 \delta(\vec{d}_S)$, where $\vec{d}_S$ is the location of the point source and $\delta(\cdot)$ is the Dirac delta distribution.

The analytical solution of equation 9 with $\vec{d}_{\mathrm{S}} = (0, 0)^{\mathrm{T}}$ is given by:

$$c(d, t) = \frac{S_0}{4\pi D} \times \gamma \left(0, \frac{d^2}{4Dt} \right) \quad , \tag{10}$$

where $d = \sqrt{x^2 + y^2}$ and $\gamma(\cdot, \cdot)$ is the lower incomplete Gamma-function. The gradient of the chemokine distribution is then given by

$$\frac{\partial}{\partial d} c(d, t) = \frac{S_0}{2\pi D} \frac{1}{d} \times \gamma \left(1, \frac{d^2}{4Dt} \right) \quad . \tag{11}$$

Note that $\gamma(1, \frac{d^2}{4Dt}) \to 1$ for $t \gg d^2/(4D)$, leading to a $1/d$ proportionality of the concentration gradient in the steady state. The $1/d$ relation enters the expression for the probability $p_{\text{sig}}(d)$ of alveolar macrophages to perform directed migration towards the AEC associated with the fungus. The maximum intensity of the signal is set at the edge of the AEC with $r_0 = 30\,\mu\text{m}$ for type I and $r_0 = 5.27\,\mu\text{m}$ for type II from its centroid. This yields the distance-dependent probability $p_{\text{sig}}(d) = r_0/d$ for $d \geq r_0$ that is used in this study.

We assume that mapping the two-dimensional planar solution of the diffusion equation to the two-dimensional spherical inner surface of the alveolus is a reasonable assumption in the limit of $r_0/(\pi r_{\text{Alv,max}}) \approx 0.076 \ll 1$.

Run-time Profiling of the Simulations

The execution time of the simulation program depends linearly on the timesteps required for the first passage of one of the alveolar macrophages. The average run-time, $t_{\mathrm{run},\Delta \mathrm{t}}$, for one timestep is independent of the spread of the first-passage-time distribution and of the scenarios (static case $\Delta t = 0.1$ min, breathing cases $\Delta t = 0.001$ min). However, this measure depends on the dynamically changing number of agents to be simulated in the timestep under consideration.

In Table S1 we show the average run-time per timestep for the different scenarios tested in this study. We see slight differences among persistent random walk and biased persistent random walk, since directions of migration have to be computed additionally in the simulations where chemotaxis is involved. Despite having spatial rescaling of all agents in the breathing scenarios in order to meet the bound-to-surface condition, run-time does not increase in comparison to the static case. The small timestep reduces the average load, since interaction events or the exit and input of AM from or into the system become rare cases. Note, however, that the overall runtime of the breathing scenarios is indeed much higher than for the static case by about two orders of magnitude, as the timesteps Δt differ by two orders of magnitude.

References Supporting Information

1. Hansen JE, Ampaya EP (1975) Human air space shapes, sizes, areas, and volumes. Journal of Applied Physiology 38: 990–5.

2. Ochs M, Nyengaard JR, Jung A, Knudsen L, Voigt M, et al. (2004) The number of alveoli in the human lung. American Journal of Respiratory and Critical Care Medicine 169: 120–4.

3. ICRP (1994) Deposition model. Annals of the ICRP 24: 36–54.

4. Balásházy I, Hofmann W, Farkas A, Madas BG (2008) Three-dimensional model for aerosol transport and deposition in expanding and contracting alveoli. Inhalation Toxicology 20: 611–21.

5. Herzog EL, Brody AR, Colby TV, Mason R, Williams MC (2008) Knowns and unknowns of the alveolus. Proceedings of the American Thoracic Society 5: 778–82.

6. Stone KC, Mercer RR, Gehr P, Stockstill B, Crapo JD (1992) Allometric relationships of cell numbers and size in the mammalian lung. American Journal of Respiratory Cell and Molecular Biology 6: 235–243.

7. Kawakami M, Takizawa T (1987) Distribution of pores within alveoli in the human lung. Journal of Applied Physiology (Bethesda, Md : 1985) 63: 1866–70.

8. Wallace W, Gillooly M, Lamb D (1992) Intra-alveolar macrophage numbers in current smokers and non-smokers: a morphometric study of tissue sections. Thorax 47: 437–440.

9. Codina R, Fox R, Lockey R (2008) Typical levels of airborne fungal spores in houses without obvious moisture problems during a rainy season in Florida, USA. Journal of Investigational Allergology & Clinical Immunology 18: 156–162.
